# The role of serum-glucocorticoid regulated kinase 1 in reproductive viability: implications from prenatal programming and senescence

**DOI:** 10.1007/s11033-024-09341-8

**Published:** 2024-03-01

**Authors:** Qiying Zhang, Ye Tian, Zhujing Fu, Shuangyu Wu, Huizhen Lan, Xuanle Zhou, Wendi Shen, Yiyun Lou

**Affiliations:** 1 Medical School, Zhejiang Chinese Medical University, Hangzhou, Zhejiang China; 2 Department of Gynaecology, Hangzhou Hospital of Traditional Chinese Medicine, Hangzhou TCM Hospital Affiliated to Zhejiang Chinese Medical University, No. 453 Tiyuchang Road, Hangzhou, 310007 Zhejiang China; 3 Jinhua Municipal Central Hospital, Jinhua, 321001 China

**Keywords:** Serum-glucocorticoid regulated kinase 1, Viability, Gametogenesis, Embryonic and fetal development, Longevity

## Abstract

**Objective:**

Organisms and cellular viability are of paramount importance to living creatures. Disruption of the balance between cell survival and apoptosis results in compromised viability and even carcinogenesis. One molecule involved in keeping this homeostasis is serum-glucocorticoid regulated kinase (SGK) 1. Emerging evidence points to a significant role of SGK1 in cell growth and survival, cell metabolism, reproduction, and life span, particularly in prenatal programming and reproductive senescence by the same token. Whether the hormone inducible SGK1 kinase is a major driver in the pathophysiological processes of prenatal programming and reproductive senescence?

**Method:**

The PubMed/Medline, Web of Science, Embase/Ovid, and Elsevier Science Direct literature databases were searched for articles in English focusing on SGK1 published up to July 2023

**Result:**

Emerging evidence is accumulating pointing to a pathophysiological role of the ubiquitously expressed SGK1 in the cellular and organismal viability. Under the regulation of specific hormones, extracellular stimuli, and various signals, SGK1 is involved in several biological processes relevant to viability, including cell proliferation and survival, cell migration and differentiation. In line, SGK1 contributes to the development of germ cells, embryos, and fetuses, whereas SGK1 inhibition leads to abnormal gametogenesis, embryo loss, and truncated reproductive lifespan.

**Conclution:**

SGK1 integrates a broad spectrum of effects to maintain the homeostasis of cell survival and apoptosis, conferring viability to multiple cell types as well as both simple and complex organisms, and thus ensuring appropriate prenatal development and reproductive lifespan.

## Introduction

A balance between cell survival and apoptosis is essential to the development of living creatures [1]. The disruption of this balance leads to the disturbance of organismal and cellular viability or even carcinogenesis [2]. Therefore, to maintain that homeostasis, an appropriate response to dynamic extracellular and intracellular challenges is pivotal to all living organisms. One such key regulatory molecule that coordinately integrates the intracellular signal transduction pathways in this response is serum-glucocorticoid regulated kinase (SGK) 1 [3, 4].

SGK1 is a serine-threonine protein kinase, belonging to the protein kinase A/protein kinase G/protein kinase C (AGC) family [2, 5]. Being highly conserved throughout eukaryotic evolution, SGK1 has already been identified in the genomes of various species [6, 7]. In addition to human, rats and mice, SGK1 is widely expressed in different species such as budding yeast (*Saccharomyces cerevisiae*) [8], *Caenorhabditis elegans* (*C. elegans*) [9] sheep [10], pig [11], monkey (*Macaca fascicularis*) [12], ground squirrel (*Ictidomys tridecemlineatus*) [13], Atlantic killifish (*Fundulus heteroclitus*) [14], spiny dogfish (*Squalus acanthias*) [15], *Mozambique tilapia* [16] and influenza virus [17]. Accordingly, SGK1 contributes to the viability of multiple cell types as well as both simple and complex eukaryotic organisms [2].

In response to various extracellular stimuli, SGK1 is transcriptionally induced and rapidly activated by fairly intricate post-translational modifications accomplished via multifarious signals. SGK1 is involved in the complex regulations of transcription and protein abundance, activating several molecules as well as epithelial ion channels and carriers [18]. Correspondingly, as a functional focal point of intracellular cross-talks, SGK1 participates in the epithelial ion transport and both cell apoptosis and survival as well [7]. In effect, SGK1 dose has been demonstrated as a crucial modulator of carcinogenesis biology [19]. Correspondingly, SGK1 enhances survival, proliferation, invasiveness, motility, epithelial to mesenchymal transition, and adhesiveness of tumor cells [20].

Since several common features shared by tumor growth and physiologic processes related to cell viability such as cell survival, proliferation and migration, malignant tumor growth and invasion exhibit striking similarities with trophoblast invasion and placental development, in particular [21]. While tumor invasion is uncontrolled, a pathological process characterized by imbalanced regulation of motility and proteolysis together with unlimited metastatic capacity, however, trophoblast invasion is a strictly regulated and self-limited, a controlled physiological event restricted in time to the implantation window, and localized in space to the endometrium and the proximal third of the myometrium [22]. Considering its “oncogenic” property [23], SGK1 presumably contributes to cell viability, particularly in prenatal programming and reproductive senescence by the same token. Intriguingly, emerging evidence points to a significant role of SGK1 in cell growth and survival, cell metabolism, reproduction, and life span, all of which reflect the energic viability of living creatures [24, 25].

The following review attempts to delineate the current, albeit limited, understanding of the physiological and pathophysiological significance of SGK1 action as well as both SGK1-dependent signal cascades and its substrates in organism viability, especially in prenatal programming and reproductive senescence.

## Methods

The databases Medline/PubMed, Web of Science, Embase/Ovid, and Elsevier ScienceDirect were searched for original articles focusing on SGK1 published (to July 2023). The search performed in Medline/PubMed to identify relevant studies has used free words “serum and glucocorticoid regulated kinase 1”, “serum and glucocorticoid inducible kinase 1”, “SGK1”, whereas searches in other databases used the terms mentioned above as keywords. Our journal database searches were not restricted by species, as we focused our review on mice and human. Reference lists of identified articles were also searched for further relevant articles. We apologize to all authors whose work could not be cited owing to space limitations.

## SGK1 involvement in gametogenesis

### SGK1 in oogenesis

The development of preovulatory follicles is specifically orchestrated by intra-ovarian growth regulatory factors, mainly estrogen and progesterone, as well as the pituitary gonadotropins, such as follicle stimulating hormone (FSH) and luteinizing hormone (LH) [26, 27]. All of these hormones are under the control of hypothalamic–pituitary–adrenal (HPA) axis [28]. SGK1 transcripts have been detected in hypothalamus and elevated postnatally [29]. In pituitary corticotrophs, SGK1 resides in the nucleus region to amplify hormone release and synchronously exert proliferative and anti-apoptotic actions via nuclear factor kappa B (NF-κB) and forkhead box binding protein 3a (FOXO3a) [30–34].

During ovarian follicular development, SGK1 mRNA selectively localized to the granulosa cells, as its induction is associated with specific stages of granulosa cell proliferation [35, 36] (Fig. 2). The expression of SGK1 is present in oocytes of primordial follicles, and is very low or even undetected in granulosa cells of preantral follicles where the cells are proliferating at an extremely slow rate [37]. As the granulosa cells differentiate to nondividing luteal cells, SGK1 in immature granulosa cells is induced rapidly by FSH and more specifically LH, physiological stimulators of granulosa cell proliferation and differentiation [35, 38]. Mediated by Sp1 binding region in the promotor of SGK1 gene, this marked increase of SGK1 expressions by FSH through the cAMP/PKA pathway is followed by a transient decline, and then reaches the maximal level as cells differentiate, coinciding temporally with the secondary burst of proliferative activity that occurs in granulosa cells of large preantral follicles as they come into preovulatory follicles [38, 39]. Richards et al. [40] have reported that Sgk1 mRNA has also been elevated in ovaries of pregnant mice. In luteal cells, FSH couples with and even impacts the IGF-I pathway via PI3K cascade, leading to an augment of SGK1 [40]. SGK is an immediate target of FSH action [41–44]. The transcription of SGK is regulated in granulosa cells of the rat ovary by FSH in a biphasic pattern. In granulosa cells cultured overnight in the absence of hormone, SGK mRNA levels were very low. The addition of FSH resulted in a rapid increase in SGK mRNA that reached a peak at 2 h and then declined by 6 h. Between 12 and 48 h of FSH, SGK mRNA levels gradually increased [43]. The LH surge rapidly initiates the terminal differentiation of granulosa cells to luteal cells. Although SGK expression is rapidly reduced by the LH surge, this decrease is transient. The expressions of SGK mRNA and protein increase as the cells begin to luteinize (within 12 h post-hCG) in vivo [44]. The levels of continue to increase as the mature corpora lutea (CL) is formed (24–48 h post-hCG) until they reach their peak during midgestation (day 15 of pregnancy) [37].

Intriguingly, subcellular distribution of SGK1 protein is related to the stage of granulosa cell function in a similar manner with unclear-cytoplasmic shuttling of SGK1 protein [37, 44]. In virtue of the adverse consequence of misinterpreting the similar stimuli, an appropriate signal propagation involves not only elaborate procedures that impart selectivity in the relay of signaling information, but also highly intricate spatial control of where signals are generated and temporal control of their duration [45]. Conceivably, the compartmentalization represents another regulation level for deciphering the signaling pathways of closely related kinases, which ensures the functional specificity even when similar phosphorylation motifs are presented [46]. With regard to SGK1, the subcellular localization relies heavily on the cell type, stage in the cell cycle, and local signaling factors [47]. Notably, SGK1 is enriched in the cytoplasm in the G_1_ phase, whereas predominantly nuclear in cells synchronously released into S phase and G_2_/M phase [3]. SGK1 has been found to shuttle between the nucleus and the cytoplasm in a cell-cycle and stimulus-dependent manner via a nuclear localization signal (NLS) that binds to nuclear import receptor importin-α [48, 49]. In granulosa cell, SGK1 responds to FSH in a biphasic manner in vitro, where this kinase is nuclear at 1 h and cytoplasmic at 48 h [37]. Subsequently, in vivo, SGK1 protein is localized to the nuclei of immature granulosa cell and some thecal cells of large preovulatory follicles, whereas the exclusively perinuclear region of the cytoplasm in luteal cells as well as some cells within the stromal compartment [39]. This distinct transition of SGK1 from the nucleus to the cytoplasm as granulosa cells cycle progression from differentiate, luteinize, cease dividing to terminal differentiated luteal cells when exiting from the cell cycle [37], indicates that SGK1 controls distinct actions in proliferative compared with terminally differentiated granulosa cells [37, 44].

Indeed, the increasing expressions and restriction to the nucleus of SGK1 in proliferating granulosa cells synergizes with the markedly down-regulation of FOXO3a [3, 4], a specific nuclear substrate of SGK1 inducing G_1_ phase cell cycle arrest to maintain ovarian follicles in long-term developmental arrest [37, 40, 47]. Meanwhile, exclusion of SGK1 from the nucleus in luteal cells favors phosphorylation of cytoplasmic targets that sustain a terminally differentiated state of luteal cells or the arrested stage within oocytes [37].

Moreover, a considerable number of reports have described the SGK1-activated channels and carriers in *Xenopus* laevis oocytes, including ENaC, voltage-gated Na^+^ channel Nav1.5 (encoded by the *SCN5A* gene), voltage-gated potassium channels KCNQ1/KCNE1, Kv 4.3 and Kv1.5, epithelial Ca^2+^ channel TRPV5 and TRPV6, Cl^−^ channel ClC-Ka and ClC-2, cystic fibrosis transmembrane conductance regulator Cl^−^ channel CFTR, amino acid transporter SN1, excitatory amino acid transporter (EAAT)1 and EAAT5, electrogenic Na^+^ coupled dicarboxylate transporter NaDC-1, posttranslational modulators PEPT2, Na^+^-phosphate cotransporter NaPiIIb, and sodium-potassium adenosine triphosphatase (Na^+^/K^+^-ATPase) [4, 31, 50–52]. All of these ion transporters regulated by SGK1 have been involved in the membrane potential nutrient, transportation and ion homeostasis of oocytes, contributing to normal oogenesis.

**Fig. 1:**
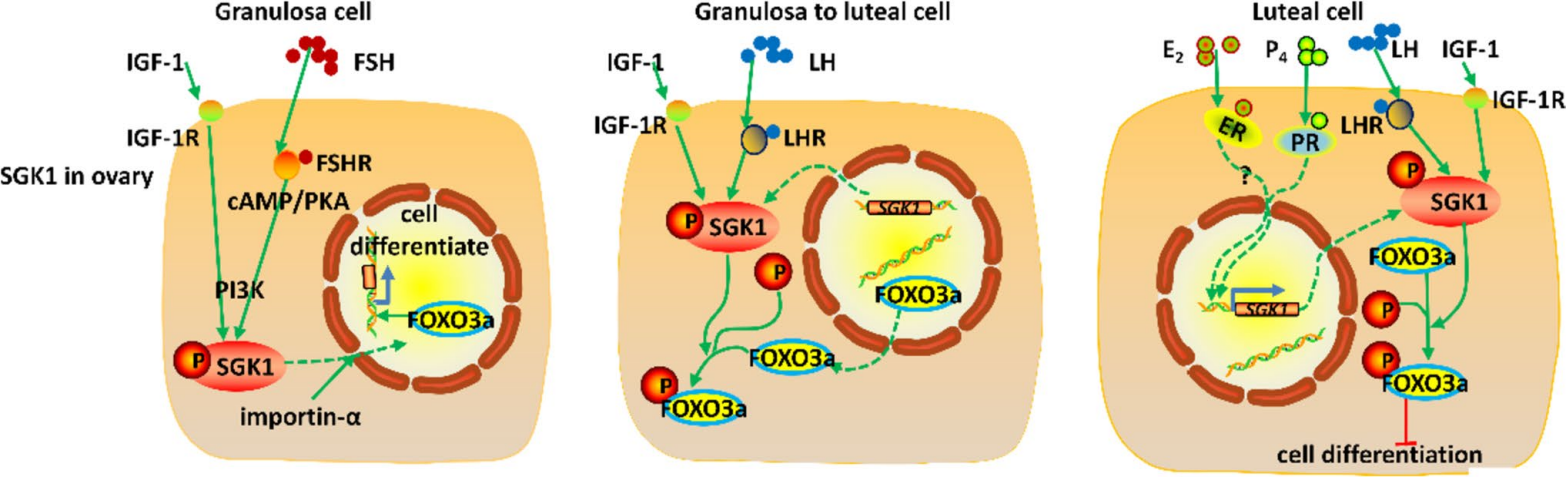
Selectively localized to the granulosa cells, SGK1 is induced by FSH, LH and steroid ovarian hormones in response to the specific stages of cell proliferation. The unclear-cytoplasmic shuttling of SGK1 protein is related to the granulosa cell differentiation

Collectively, the hormonally regulated expressions of SGK1 mRNA and the dramatic switch in the subcellular localization of SGK1 protein in proliferating granulosa cells, as well as in terminally differentiated luteal cells and resting oocytes (Fig. 1) [53], indicate that this kinase is involved in controlling cell cycle progression and stimulating differentiation throughout follicular development and luteinization [37, 44, 53].

### SGK1 in spermatogenesis

In males, on the other hand, both high-quality sperm production and normal testicular volume require normal function of FSH [54]. Accordingly, FSH-inducible transcriptions of SGK1 are also present in spermatogenic cells [55]. The blood–testis barrier (BTB) is a complex protein barrier structure between the blood vessels in the testis and the seminiferous tubules, which plays an important role in the process of spermatogenesis [56]. Studies have shown that SGK1 localizes to the acrosome during the course of epididymal maturation, and could be involved in endowing sperm with basal motility by phosphorylating and inactivating of glycogen synthase kinase 3 (GSK3) [55] and downregulating the abundance of β-catenin protein in the BTB [57]. Other studies have shown that SGK1 promotes the proliferation and anti-apoptosis of immature SCs [58] through PI3K, PDK1, AKT signaling pathways [59, 60], regulates the protein synthesis and cytoskeleton of mature SCs to change the structure of BTB [60], and promotes spermatogenesis. In addition, SGK1 highlights the role of FoxO3a in regulating oxidative stress [61], which can damage BTB by destroying BTB-associated junction proteins and inducing apoptosis of Sertoli cells, leading to testicular damage and spermatogenesis disorders [62], which could lead to male infertility [63].

Of note, one downstream substrate of SGK1, cystic fibrosis transmembrane conductance regulator (CFTR) has been implicated in sperm transportation and capacitation [64]. Thus, it is postulated that the upstream regulator SGK1 may also be involved in sperm maturation. This speculation awaits further investigation.

## SGK1 involvement in embryonic development

As a transitory epiblast marker [65], proper expression of SGK1 is involved in early embryonic specifications. SGK1 has been detected at very early stages of embryogenesis [66, 67]. In the developing genitals and forelimb buds, differential expressions of SGK1 are dependent on mammalian *HOXD*, a gene encoding transcription factors that are essential to morphogenesis along the multiple body axes [68]. SGK1 expression was reported to be largely limited to the proximal portion of the interdigital zone of mouse embryo and abundant within the distal ventral mesenchyme of developing genital bud as well, both of which are immediately adjacent to domains of apoptosis [68]. In accordance with the smaller size of appendages observed in the mouse of *HoxD* mutant, deregulation of SGK1 could contribute to cell apoptosis in both structures [68], probably by phosphorylating and inactivating the pro-apoptotic downstream target FOXO3a (FKHRL1) [32]. Moreover, genetic inactivation of SGK1 contributes to the excessive apoptosis in the ectoderm [1]. In the endoderm or dorsal mesoderm, PI3K-dependent activation of SGK1 is speculated to ensure ectodermal cell survival during early *Xenopus* embryogenesis in an intercellular signaling cascade, which is mediated by decreasing death receptors and components of the death-inducing signaling complex (DISC) [1]. In this extrinsic pathway, SGK1 stimulates NF-κB signaling, leading to the accumulation of bone morphogenetic protein 7 (BMP7), which in turn acts on neighboring or distant ectodermal cells to repress the expressions of genes encoding DISC components in the ectoderm and thus promote cell survival across germ layers during amphibian embryonic development [1]. The study found that two growth factors, bone morphogenetic protein 2 (BMP-2) and transforming growth factor-β (TGFβ), significantly increased both Nuclear factor of activated T cells (NFAT)-5 protein expression and transactivation in nucleus pulposus cells located at the center of the intervertebral disc. This, in turn, affects disk cell function through chondroitin sulfate and aggrecan synthesis [69]. And NFAT5 was known to increase the expression of SGK1 [70]. Additionally, BMP15 and GDF9 are expressed by oocytes and play a key role in granulosa cell development and fertility [71]. During follicular development, the induction of SGK1 is closely related to the specific stage of granulosa cell proliferation [36]. BMP15 and GDF9 promote FSH-induced progesterone synthesis in preovulatory follicular granulosa cells [72], and significantly increase the expression of SGK1 under the mediation of Sp1 [38, 73]. Thus, BMP2, BMP7, BMP15 and GDF9 play a role in the regulation of SGK1. Of note, during the earliest stages of *C. elegans* embryogenesis, SGK1 was found to antagonize *skn1*, a transcription factor that orchestrates gene expression programs involved in the establishing development of the mesendoderm [74]. Thus, as a primary downstream effector of rapamycin-insensitive companion of TOR (rict-1)/TORC2 activity, SGK1 knockdown partially rescues *skn1* deficient-associated lethality by restoring mesendodermal specification [36, 74]. Apart from the obvious species difference, the discrepancy in these findings was explained by specific pathways involved in and different states of embryogenesis. Nonetheless, these data point to an essential role for SGK1 in embryogenesis.

Interestingly, merging evidence reveals that SGK1 is pivotal to the early vasculogenesis [51, 75, 76]. As early as embryonic day 8.5 (E8.5), SGK1 was already observed throughout mouse embryo, especially higher in the yolk sac [77], a primary tissue where angioblasts differentiate into endothelial cells in the blood islands to form the earliest cardiovascular system [75]. Ablation of SGK1 results in early cardiovascular failure and embryonic lethality at E10.5–E11.5 due to defective angiogenic remodeling and to impaired myocardial trabeculation [75, 76]. The lethal effects of SGK1 absence are accompanied by decreased expression levels of Notch signaling components [75]. Moreover, phosphorylation of NDRG1 by SGK1 alters NF-κB signaling and the expression of the downstream target VEGF-A, a pivotal growth factor plays a pivotal role in mediating the differentiation of endothelial cells [51]. The disruption of SGK1 leads to defective endothelial cell migration and tube formation, while increased apoptosis of vascular smooth muscle cells and endothelial cells, thus suggesting a pro-survival role for SGK1 during angiogenesis in the cardiac neo-angiogenesis and the endocardium-myocardium crosstalk during trabeculation [51, 75, 76, 78].

**Fig. 2:**
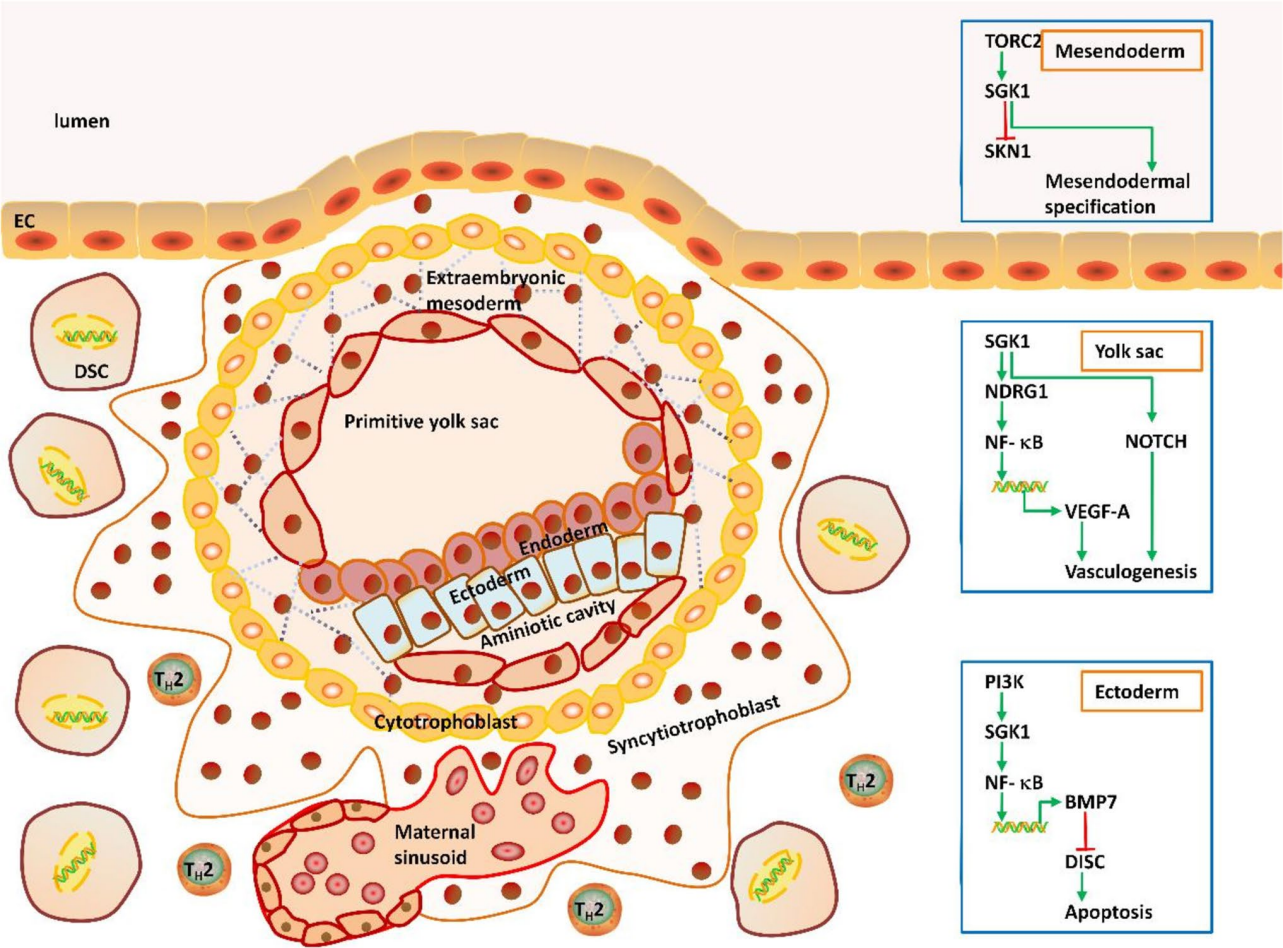
Positive effects of SGK1 in embryogenesis. SGK1 promotes cell survial and early vasculogenesis to participate in the embryonic development. The red arrows with flat heads denote inhibitory modification. *EC* epithelial cell; *DSC* decidual stromal cell; *T*_*H*_2 type 2 helper T cell; *TORC2* target of rapamycin complex 2; *SGK1* serum and glucocorticoid regulated kinase 1; *NDRG1* N-myc downstream-regulated gene 1; *NF-κB* nuclear factor kappa-light-chain-enchaner of activated B cell; *VEGF-A* vascular endothelial growth factor A; *NOTCH* Notch signaling pathway; *PI3K* phosphatidylinositide 3-kinases; *BMP7* bone morphogenetic protein 7; *DISC* death-insucing signaling complex; *P* phosphate

Together, SGK1 participates in regulating embryonic development by promoting cell survival and early angiogenesis (Fig. 2).

## SGK1 involvement in fetal development

Along with fetal development, the expression of SGK1 is stringently regulated in a temporal context [77]. By whole-mount in situ hybridization of mouse embryo, Lee et al. [77] have observed that SGK1 is prominent in the yolk sac and decidua at E8.5, and then highly expressed in the lung buds, blood vessels surrounding the somites, developing otic vesicle and the heart chamber during developmental stages E9.5–E12.5. By days E13.5–E16.5, SGK1 is extensively localized in the brain stem, distal epithelium and the thymus, terminal bronchi/bronchioles, adrenal gland, liver and intestines [77, 79]. And then the expression of SGK1 transcripts remains high in mouse heart tissue, but delinks in the other embryonic tissues on the later stages [77].

This tissue- and stage-specific expression is highly congruous with the pluripotent role of SGK1 in the transition from intrauterine to extrauterine life [77, 80]. Particularly, as a crucial regulator of ion channels and transporters [30, 81], SGK1 has been proposed in fetal volume regulation and amniotic fluid secretion, the physical processes attributed specifically to the lung, kidney and small intestine [80]. The induction of SGK1 increases distal lung fluid absorption by activating α subnet of epithelial Na^+^ channel (α-ENaC) [82] through Nedd4-2 suppression in fetal lung tissue [83]. By the same token, SGK1 in placental trophoblast contributes to normal sodium homeostasis in the bidirectional process of maternal–fetal fluid exchange by modulating trophoblast Na^+^ transport under the stimulation of corticosteroids [84]. Besides, two single-nucleotide polymorphisms (SNPs) of SGK1 (rs1057293 and rs1743966) were reported to be related to transient tachypnea of the newborn (TTN) [85], proposing a protecting role in fetal growth and development [86]. Consistently, SGK1 protein is significantly lower postnatally, while increasing prominently in the fetal lung [80]. By comparison, SGK1 protein in renal cortex is rather steady for not increasing further with gestational age or even after birth [80]. In late gestation, the increased expressions of SGK1 in brain stem, hypothalamus, and hippocampus, reveal the development of the fetal brain–pituitary–adrenal axis [79].

Taken together, SGK1 has a positive effect on the prenatal development. Intriguingly, maternal SGK1 expression is speculated as a modifier of intrauterine fetal development [53]. In the placenta of intrauterine programmed gestational diabetes mellitus (GDM) rats, increased phosphorylation of SGK1 combined with other alterations in mTOR signaling apparently lead to fetal overgrowth in female offspring by inducing a pro-oxidant and pro-inflammatory intrauterine environment [87, 88]. Concordantly, the offspring of wild type (SGK1^*+*/*+*^) female mice mated with SGK1 knockout (SGK1^*−*/*−*^) male mice had significantly higher blood pressure and slower weight gain postnatally following prenatal fasting, whereas the offspring of SGK1^*−*/*−*^ mothers had no such phenotypes [31]. Therefore, maternal signals mediated by SGK1 participate in the fetal programming of diabetes and hypertension, shedding light on the decisive role of SGK1 in the maternal side of fetal programming.

## SGK1 involvement in reproductive senescence

Upon DAVID bioinformatics analysis in *C. elegans* gene, SGK1 has been identified to delay reproductive senescence [89]. Accordingly, loss of SGK1 in *C. elegans* results in defective egg-laying, extended generation time, reduced fecundity [90]. Moreover, beyond prolonging reproductive lifespan, inactivation of SGK1 has been proposed to increase healthy life expectancy, rather than altering total lifespan [89]. Thus, it seems that reproductive aging could have a predominant effect on a healthy somatic lifespan [89].

Therefore, reproductive senescence has been suggested as a distinguishing feature of aging, noted by a progressive age-related decline in fertility mainly attributed to the reduction in oocyte quantity as well as quality [89, 91]. No relevant study has demonstrated that SGK 1 can cause premature ovarian failure or luteal insufficiency, however, SGK1 alterations can lead to reproductive dysfunctions. According to reports, excessive SGK1 in the luminal epithelium of the endometrium may disturb the delicate uterine luminal fluid (ULF) balance, leading to a hostile environment that prevents embryo implantation. Additionally, it may dysregulate endometrial receptivity genes, contributing to unexplained implantation failure in humans [31]. Since the risk of infertility, abortion and birth defects increased along with germline ages, the somatic health should be well sustained to support reproductive activities [89]. Consistent with this view, the most significant prognosticator for reproductive senescence is the maternal age [91].

Over the past two decades, studies in model organisms, such as worms, yeast, and mice, have witnessed a rapid progress in identifying avenues related to senescence [92, 93]. For example, decreasing insulin and insulin-like growth factor-1 signaling (IIS), restricting food intake (dietary restriction or DR), reducing germline function, slowing mitochondrial respiration, or lowering temperature can all extend lifespan [94]. Since many aspects of the molecular regulation of senescence are conserved from worms to mammals [93], the *C. elegans* model of senescence sheds light on the regulation of reproductive senescence and lifespan in other organisms, including humans [95–97].

Given the pronounced role in cell survival, proliferation, and growth that mentioned above, SGK1 has been implicated to be involved in the modulations of reproductive senescence [89] and lifespan of *C. elegans* [98]. Ample evidence suggests that IIS is central to whole-organism metabolism, development, and stress response, and has a conserved role in senescence and lifespan [94, 99, 100]. In *C. elegans*, mutations in the *DAF-2* gene (encodes the *C. elegans* homolog of the mammalian insulin/IGF-1 receptors) increase stress resistance and longevity [101, 102]. In DAF-2 insulin signaling, TORC2, with its specific component Rictor, has been firmly implicated in senescence and regulation of cellular energetics, growth and metabolism [74]. Intriguingly, the effects of rict-1/TORC2 were largely mediated through SGK1 in *C. elegans* [74]. Loss-function of SGK1 results in an overall reduced brood size, developmental delay, defective egg-laying, extended generation time, increased stress resistance and an extension of lifespan, thereby phenocoping the phenotypes associated with mutation of upstream rict-1/TORC2 [74]. The effect of reduced SGK1 is mainly achieved through de-suppression of downstream effector DAF-16/FOXO, an IIS-inhibited FOXO homolog regulating reproduction, growth and lifespan in *C. elegans* [103–106]. SGK1 directly phosphorylates DAF-16/FOXO proteins, thus promoting its cytosolic localization [90, 107], whereby controlling development, stress response, and longevity through rict-1/TORC2 signaling cascade [108].

Similarly, the antioxidant-mediated longevity has been also found to activate the SGK1/DAF-16 pathway [109]. In this context, SGK1/DAF-16 has been reported to impact the generation of reactive oxygen species (ROS) [109], which accounts for ovarian senescence as well as poor oocyte quality [110].

Moreover, loss of germline proliferation regulates the lifespan within the SGK1-dependent DAF-2-DAF-16 pathway [103]. Ablation of mitotic germ cells leads to nuclear entry of intestinal DAF-16 and extends lifespan [106]. Alternatively, SGK1 in tissues other than hypodermis inhibits DAF-16 activity that counteracts hypodermal DAF-16 [111]. Activated somatic DAF-16 in the epidermis shortens lifespan by promoting a tumorigenic germline phenotype and disrupting the surrounding extracellular matrix of *C. elegans* [111]. Thus, SGK1 has been indicated in the functional balance of tissue-specific DAF-16 activities, exerting an antagonistic regulation on lifespan [111].

On the other side, SGK1 may delay senescence by regulating proteins other than DAF-16/FOXO, such as transcription factor SKN-1 [74, 112]. In this regard, SGK1 not only inhibits DAF-16 protein but also directly inactivates SKN-1 in parallel [113]. SKN-1 has conserved functions in stress detoxification, cellular homeostasis, metabolism and longevity [114]. SGK1 phosphorylates SKN-1 and prevents its accumulation in intestinal nuclei, thus inhibiting its functions on lifespan [74]. Interestingly, SKN-1/Nrf establishes the development of the mesendoderm in embryos as illustrated above [74]. Therefore, TORC2/SGK1 signal cascade influences in the context of both embryonic development and senescence [74]. Additionally, SGK1 negatively regulates development and reproduction by decreasing the dosage compensation complex (DCC) activity via physically interacting with its component DPY-21 protein post-embryonically [115].

Taken together, SGK1 may orchestrate gene expression programs involved in growth and metabolism cellular homeostasis, thus contributing to delayed reproductive senescence and prolonged lifespan [115]. Since most of these data are from *C. elegans* concentrating on aging, the role of SGK1 in regulating reproductive senescence in human merits further investigation.

## Conclusion

Since inundated with a diverse array of extracellular stimuli, overwhelming data has landed SGK1 center stage of numerous signaling pathways integrating several cellular processes important to the organism viability, including cell survival and/or apoptosis, cell proliferation and growth, cell migration and differentiation, nutrients and energy conditions with extracellular signals [3, 4, 116, 117]. Significantly, as a focal point of several signaling outputs, SGK1 is deeply involved in the regulation of prenatal programming, such as gametogenesis, embryonic and fetal development, and reproductive life span as well via osmoregulation and balancing anti- or pro-apoptotic actions [31]. Moreover, this anti-apoptotic virus of SGK1 shows a gender-specific expression pattern [47]. Consistent with this view, SGK1 seems to play a more prominent role in endometrial regeneration and maternal-fetal interface integrity [31]. However, SGK1 abrogation does not fully disrupt the respective functions [118]. This discrepancy might attribute partly to the differences in experimental approaches and array platforms. Nevertheless, current knowledge spurs the need to further investigate whether SGK1 is an innocent bystander or a major driver actively participating in the molecular mechanisms [20] that stimulates cell proliferation and survival, thereby conferring organism viability.

## Data Availability

No data associated in the manuscript.

## Abbreviations

SGK: Serum-glucocorticoid regulated kinase
AGC: The protein kinase A/protein kinase G/protein kinase C
*C. elegans*: *Caenorhabditis elegans*
FSH: Follicle stimulating hormone
LH: Luteinizing hormone
HPA: Hypothalamic–pituitary–adrenal
NF-kB: Nuclear factor kappa B
FOXO3a: Forkhead box binding protein 3a
NLS: Nuclear localization signal
EAAT: Excitatory amino acid transporter
GSK3: Glycogen synthase kinase 3
CFTR: Cystic fibrosis transmembrane conductance regulator
DISC: Death-inducing signaling complex
BMP7: Bone morphogenetic protein 7
E8.5: Embryonic day 8.5
SNPs: Single-nucleotide polymorphisms
TTN: Transient tachypnea of the newborn
GDM: Gestational diabetes mellitus
IIS: Insulin-like growth factor-1 signaling
DR: Dietary restriction
ROS: Reactive oxygen species
BTB: Blood–testis barrier
CL: Corpora lutea
NFAT: Nuclear factor of activated T cells
